# Enzootic mosquito vector species at equine encephalitis transmission foci in the República de Panamá

**DOI:** 10.1371/journal.pone.0185491

**Published:** 2017-09-22

**Authors:** Rolando Torres, Rafael Samudio, Jean-Paul Carrera, Josue Young, Ricardo Márquez, Lisbeth Hurtado, Scott Weaver, Luis Fernando Chaves, Robert Tesh, Lorenzo Cáceres

**Affiliations:** 1 Instituto Commemorativo Gorgas de Estudios de la Salud, Ciudad de Panamá, República de Panamá, Departmento de Entomología Medica; 2 Mastozoological Society of Panamá, Ciudad de Panamá, República de Panamá; 3 Instituto Commemorativo Gorgas de Estudios de la Salud, Ciudad de Panamá República de Panamá, Departmento de Genomica y Proteomica; 4 Instituto Commemorativo Gorgas de Estudios de la Salud, Ciudad de Panamá, República de Panamá, Departmento de Análisis Epidemiológico y Bioestadísticas; 5 Institute for Human Infections and Immunity and Department of Pathology, University of Texas Medical Branch, Galveston, TX, United States of America; 6 Programa de Investigación en Enfermedades Tropicales (PIET), Escuela de Medicina Veterinaria, Universidad Nacional, Heredia, Costa Rica; 7 Centro de Investigación en Enfermedades Tropicales (CIET), Universidad de Costa Rica, San Pedro de Montes de Oca, Costa Rica; University of California Davis, UNITED STATES

## Abstract

The identification of mosquito vector species present at arboviral enzootic transmission foci is important to understand transmission eco-epidemiology and to propose and implement prevention and control strategies that reduce vector-borne equine encephalitis transmission. The goal of this study was to identify mosquito species potentially involved in the transmission of enzootic equine encephalitis, in relation to their abundance and diversity at three endemic regions in the República de Panamá. We sampled adult mosquitoes during the dry and rainy season of Panamá. We employed CDC light traps with octanol, EV traps with CO_2_ and Trinidad 17 traps baited with live hamsters. Traps were deployed in the peridomicile and extradomicile of houses from 18:00 to 6:00 h. We estimated the abundance and diversity of sampled species. We collected a total of 4868 mosquitoes, belonging to 45 species and 11 genera, over 216 sampling nights. *Culex (Melanoconion) pedroi*, a major Venezuelan equine encephalitis vector was relatively rare (< 2.0% of all sampled mosquitoes). We also found *Cx*. *(Mel) adamesi*, *Cx*. *(Mel) crybda*, *Cx*. *(Mel) ocossa*, *Cx*. *(Mel) spissipes*, *Cx*. *(Mel*) *taeniopus*, *Cx*. *(Mel) vomerifer*, *Aedes scapularis*, *Ae*. *angustivittatus*, *Coquillettidia venezuelensis*, *Cx*. *nigripalpus*, *Cx*. *declarator*, *Mansonia titillans*, *M*. *pseudotitillans* and *Psorophora ferox* all species known to be vectorially competent for the transmission of arboviruses. Abundance and diversity of mosquitoes in the sampled locations was high, when compared with similar surveys in temperate areas. Information from previous reports about vectorial competence / capacity of the sampled mosquito species suggest that sampled locations have all the elements to support enzootic outbreaks of Venezuelan and Eastern equine encephalitides.

## Introduction

New World alphaviruses, like Venezuelan (VEEV) Eastern (EEEV) and Western equine encephalitis virus (WEEV), are etiologic agents of major zoonotic diseases transmitted by mosquitoes that affect humans and equines [1]. The equine encephalitides are often lethal or leave severe neurological sequelae following periodic epizootics and epidemics. Therefore, these diseases have mandatory reporting to the World Organisation for Animal Health, OIE [2]. To date there are no safe and efficient vaccines against the infection by an alphavirus [2–4]. VEE is considered the most important re-emerging zoonosis affecting hundreds of thousands of equines and humans through the Americas [4–6]. Meanwhile several EEE outbreaks have affected equines and humans, with a high mortality rate and significant neurologic damage in surviving individuals [7].

VEEV is a RNA virus belonging to the Togaviridae family and the *Alphavirus* genus [8]. VEEV is a diverse virus where specific subtypes have been associated with the epidemic/epizootic cycle, IAB and IC, which have been frequently isolated in human and equine epidemics, associated with zoophilic vectors [9]. It remains an open question how these subtypes are maintained during the inter-epizootic periods and the role of vectors in such periods, highlighting the need for a better understanding of vector species diversity in transmission areas [10–12]. By contrast, subtypes ID, IE, IF and II-VI have been associated with endemic and enzootic transmission in tropical and subtropical sylvatic areas. These subtypes can be easily isolated from mosquito vectors and small vertebrate reservoir hosts [13] and are becoming increasingly associated with human cases [14]. For example, subtype ID is very common across the República de Panamá and all over Central America, Colombia, Venezuela, Mexico and the USA [8,15–18]. In Panamá subtype IE, following its 1962 isolation from Almirante in Bocas del Toro province [19], has never been isolated again.

EEE epizootics have been recorded in Panamá since 1936 [20]. In 1986 a well-documented EEE epizootic outbreak occurred in Panamá, mainly affecting horses, during the rainy season. This outbreak was simultaneous with bird migrations from North to South America [21]. The most recent well documented EEE outbreak in Panamá occurred in 2010 in Darién, where VEEV was also being transmitted [6]. In this outbreak, there were 19 human encephalitis cases of which 7 were infections by EEEV, 3 by VEEV, one case was a co-infection by VEEV and EEEV, while 3 patients died [4].

Dominant VEEV vectors include *Aedes taeniorhynchus* (Wiedemann, 1821) and *Psorophora confinnis* (Lynch & Arribalzaga, 1821), which have been associated with epizootic VEEV transmission [22,23]. Enzootic transmission is believed to be almost exclusively carried out by the *Spissipes* section of the *Melanoconion* subgenus of the *Culex* genus [15,24]. VEEV has been isolated from *Cx*. (*Mel*) *portesi* (Sevenet and Abonnenc, 1941) which transmits Mucambo virus (VEEV subtype IIIA) in Trinidad; *Cx*. (*Mel*) *cedeci* (Stone & Hair, 1968) which transmits Everglades virus (VEEV subtype II) in Southern Florida, USA; *Cx*. (*Mel*) *aikenii* (Aiken & Rowland, 1906) *sensu lato ocossa* and *panocossa* which transmits subtype ID in Panamá and *Cx*. (*Mel*) *taeniopus* (Dyar & Knab, 1907) which transmits subtype IE and is the main VEEV vector in Guatemala [8,9,11,23,25].

In Panamá, from the time of the first enzootic VEEV isolation (subtype ID a.k.a., strain 3880) from a fatal human case [26], frequent endemic and enzootic outbreaks have been described via virus isolation from mosquito vectors, rodent reservoirs, equines and humans [15]. The recent cyclic and explosive enzootic and epizootic VEE outbreaks in countries neighboring Panamá, in addition to the frequent isolation of enzootic subtype ID VEEV from the Darién province and other regions in Panamá [4,6] calls for a better knowledge of the mosquito fauna, especially the identification of potential VEEV vectors. Here, we will define a potential vector as a species which has been found infected by a pathogen without a bloodmeal in a previous field study elsewhere or which has been experimentally shown as competent to transmit the pathogen in the laboratory [13]. In this study we present results from a series of mosquito surveys in three regions with a history of equine encephalitis transmission, placing an emphasis on the diversity and abundance of potential VEEV and EEEV vectors.

## Materials and methods

### Study site

**Fig 1:**
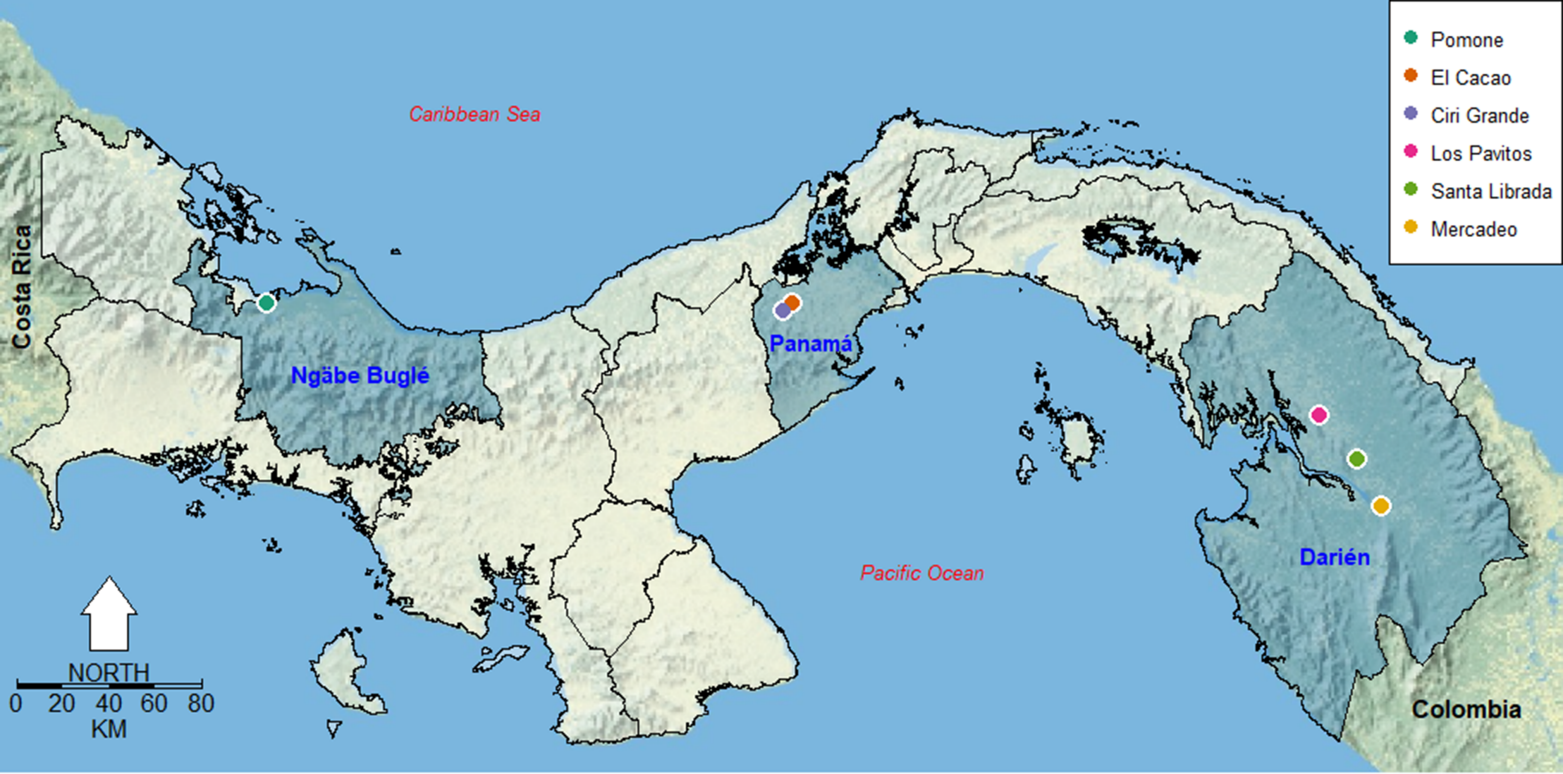
Map of study locations in the República de Panamá. The three provinces where the locations were situated are highlighted and have their names indicated in blue (In Panamá province we only highlight Western Panamá province). The inset legend shows the color code for the six study locations. This map was made using the open source software R using as a background a public domain map from the US National Park Service (https://www.nps.gov/hfc/carto/data-sources.cfm).

We designed this study to compare mosquito species composition from three enzootic arbovirus (VEEV and EEEV) transmission foci in the Panamá and Darién provinces and the autonomous indigenous Comarca (territorial political division assigned to indigenous groups) Ngäbe Buglé. In the selected study areas infections in humans, horses and/or wildlife animals have been reported [4,6], or in mosquito pools identified to the genus level [27]. Darién is the easternmost province in Panamá, bordering Colombia. The natural landscape is dominated by tropical rainforest and the climate is tropical with an extended dry season. Total annual rainfall is over 2500 mm, with one or two dry months with less than 60 mm. Temperature ranges between 18 and 23°C around the year [28]. In this province we selected the following locations for mosquito sampling: Mercadeo with 36 households and 206 inhabitants, Santa Librada with 170 households and 300 inhabitants and Los Pavitos with 30 houses and 95 inhabitants. Western Panamá Province has a warm pre-mountain humid tropical rainforest. Annual rainfall adds to 1571 mm, with a mean annual temperature of 26.5°C [29]. Here, we selected El Cacao and Ciri Grande as sampling locations. Ngäbe Buglé is also covered by premountain tropical rainforest and has an annual rainfall around 400 mm and mean temperatures around 25°C year-round. Here, we collected mosquitoes at Pumona. In all the sampling locations it is worth highlighting that the landscape is very homogeneous from the standpoint of ecological disturbances, since at the local scale of our sampling locations there was a similar mix of forest and cattle farming grounds near households. In all studied locations the main economic activities are cattle farming, wood extraction and subsistence agriculture. Fig 1 is a map showing the sampling locations.

### Mosquito sampling

**Fig 2:**
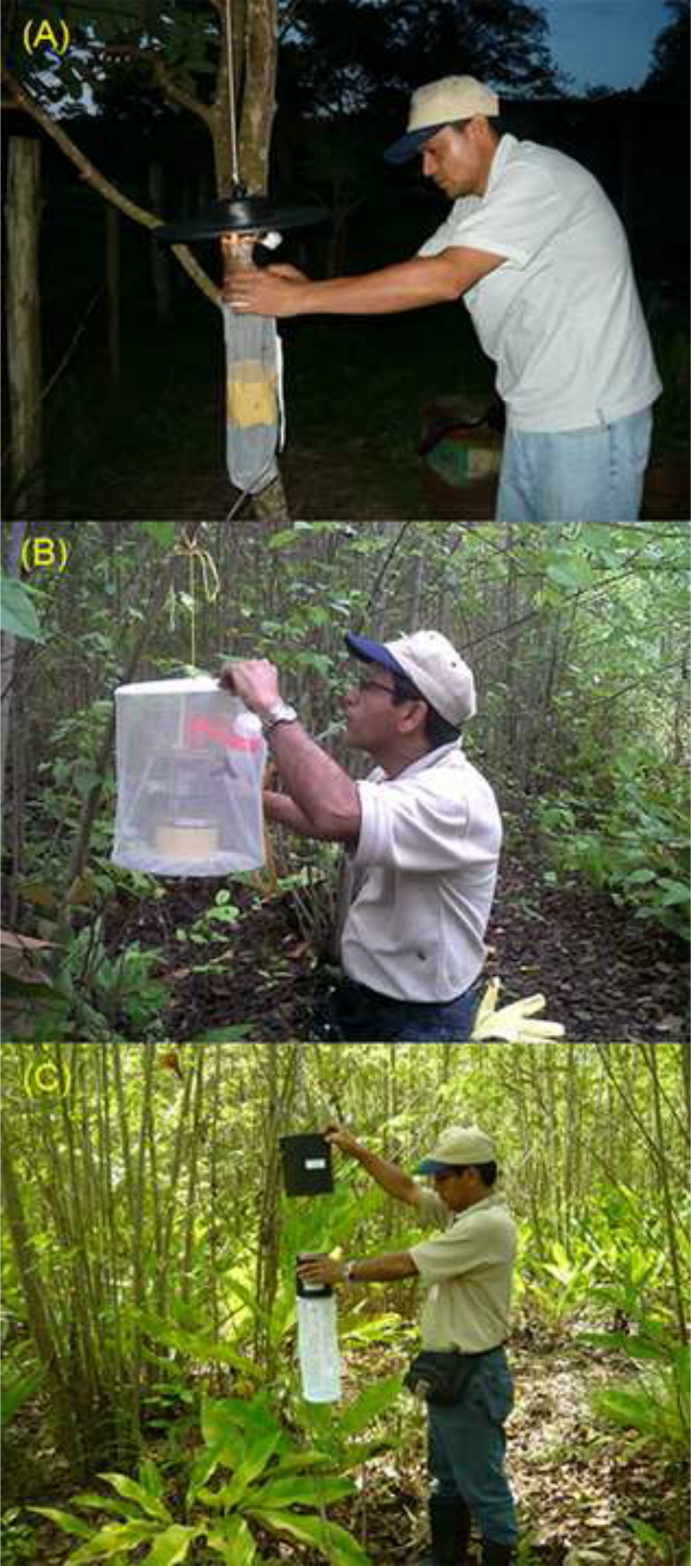
Mosquito TRAPS. (A) CDC light with Octanol, (B) Modified Trinidad 17, baited with one hamster and (C) EVS baited with CO_2_.

At each sampling location we put three kinds of traps over three consecutive nights (18:00 to 6:00) at 1.5 m above the ground in peridomiciliary areas and forests near to the houses, hereafter referred as extradomicile [30,31]. In each locality we employed 10 CDC light traps baited with octanol (Fig 2A), eight modified Trinidad 17 (TT-17) traps (Fig 2B), baited with one live hamster and eight EVS traps (Fig 2C) baited with CO_2_ [25]. Sampling was done during February-March (dry season) and September-October (rainy season) of 2011 and 2012, trying to sample species from both the dry and rainy season. Collected mosquitoes were killed, by flash-freezing, soon after collection and identified at the genus level in the field. Samples were then placed in plastic vials by trap type and sampling date and stored in liquid nitrogen before transportation to the Departamento de Entomología Médica at the Instituto Conmemorativo Gorgas de Estudios de la Salud, where identification at the species level, whenever possible, was performed using taxonomic keys [24,32,33] and the reference collection at the Institute.

### Data analysis

We used mosquito species abundance data to estimate mosquito species relative abundance and diversity using the software EstimateS, 8.2.0™ [34]. We specifically estimated the Simpson and Shannon-Wiener diversity indices to compare patterns of diversity at each study site. The choice of these two indices was done given the emphasis of the former on dominant species, as opposed to the latter which focuses on the whole community [35]. We also estimated species richness by counting the number of species at each site and by estimating the Margalef index. Finally, we estimated species similarity between sampling locations using the Sorensen index. We also estimated the median abundance and its SE for females of all collected taxonomic units. For the analyses we used the additive mosquito counts, from all three types of traps, for each mosquito taxonomic unit. In all the analyses we considered taxonomic units identified at the genus level as a distinct species, since they likely included, in all cases, individuals belonging to species not identifiable with morphological keys.

### Ethical clearance

No permits were required since humans were not involved in the study. Use of hamsters was approved by the “Comité Institucional de Uso y Cuidado de Animales de Laboratorio” (CIUCAL) of Instituto Conmemorativo Gorgas de Estudios de la Salud, in accordance with law No. 23 of January 15 1997 (Animal Welfare Assurance) of República de Panamá, as presented within the research protocol of project “Estudio del subtipo ID del complejo de Encefalitis Equina Venezolana en Panamá”.

### Materials and data availability

All data analyzed in the results section are presented within the text of this article. Voucher specimens of collected mosquito species are available at the Colección de Insectos, Departmento de Entomología Medica, Instituto Conmemorativo Gorgas de Estudios de la Salud.

## Results

For each of the six study locations we sampled a total of 36 nights, totaling 216 sampling nights across all study sites. We collected a total of 4868 mosquitoes belonging to the following 11 genera: *Aedes*, *Anopheles*, *Aedeomyia*, *Coquillettidia*, *Culex*, *Deinocerites*, *Haemagogus*, *Mansonia*, *Psorophora*, *Uranotaenia* and *Wyeomyia*. From these 11 genera we were able to identify 45 mosquito species, and a total of 61 taxonomic units (Table 1). The most abundant species was *C*. *venezuelensis* (Theobald), 1912) 22.2%, followed by *Cx*. *(Mel) dunni* (Dyar, 1918) 4.0%, *Ae*. *angustivitatus* (Dyar & Knab, 1907) 2.6%, *Ps*. *cingulata* (Fabricius, 1805) 2.5%, *Cx*. *(Mel) pedroi* (Sirivanakam & Belkin, 1980) 2.0%, *Ps*. *confinnis* (Theolbold, 1887) 2.0% and *U*. *lowii* (Theobald, 1901) 1.8%, *Cx*. *declarator* (Dyar and Knab, 1906) 1.2%, *Cx*. *(Mel) spissipes* (Theobald, 1903) 1.1%, *An*. *neomaculipalpus* (Curry, 1933) 1.1% and *Ae*. *fulvus* 1.0%. All other species accounted for less than 1.0% of the total sample. The most species rich region was Darién with 52 taxonomic units, followed by Ngäbe Buglé with 23 taxonomic units and Panamá with 21 taxonomic units. *C*. *venezuelensis* (Table 1) was captured in all sampling localities, being the most abundant in Mercadeo (54.9%) and Pumona (42.3%), and less abundant in the other localities: Santa Librada (1.6%), Los Pavitos (0.6%), El Cacao (0.5%) and Ciri Grande (0.1%). In contrast, *Cx*. *(Lutzia) allostigma* (Howard, Dyar & Knab, 1915), *Ps*. *ferox* (Humboldt 1819), *Wyeomya chalcocephala* (Dyar & Knab, 1906) and *W*. *hosauto* (Dyar & Knab. 1907) were only collected at single locations.

**Table 1:** Mosquito species composition and abundance in three regions with equine encephalitis transmission in the República de Panamá. Data are presented as total by region. The sampling trap-nights effort is indicated by **n**. Please, note that sampling effort was the same at the location level, and differences in **n** reflect differences in the number of sampled locations by region.

Species	Regions					
	Darién		Panamá		Ngäbe Buglé	
	Median Abundance	±SE	Median Abundance	±SE	Median Abundance	±SE
	n = 108		n = 72		n = 36	
*Ae*. *angustivittatus*	42.00	51.70	0.00	0.00	5.00	5.70
*Ae*. *fulvus*	221.00	0.00	0.00	0.00	0.00	0.00
*Ae*. *scapularis*	12.70	16.50	4.00	0.00	0.00	0.00
*Ae*. *serratus*	10.70	11.50	0.00	0.00	2.00	0.00
*Aedes spp*.	7.00	0.00	0.00	0.00	6.00	0.00
*Aedes (Ochlerotatus) spp*.	26.00	31.10	0.00	0.00	6.00	0.00
*Aedes (Finlaya) spp*.	1.33	1.87	0.00	0.00	7.00	1.41
*Aedeomyia squamipennis*	101.00	99.00	0.00	0.00	0.00	0.00
*Anopheles albimanus*	0.00	0.00	0.00	0.00	5.00	2.83
*An*. *apicimacula*	6.00	0.00	3.00	0.00	4.50	2.50
*An*. *malefactor*	5.00	0.00	0.00	0.00	0.00	0.00
*An*. *neomaculipalpus*	3.00	2.00	0.00	0.00	47.50	4.95
*An*. *oswaldoi*	3.00	0.00	0.00	0.00	0.00	0.00
*An*. *pseudopunctipennis*	4.00	0.00	15.00	0.00	0.00	0.00
*An*. *punctimacula*	7.50	2.50	1.00	0.00	0.00	0.00
*An*. *strodei*	0.00	0.00	2.00	0.00	8.50	0.71
*An*. *triannulatus*	4.50	2.50	0.00	0.00	0.00	0.00
*An*. *(Anopheles) spp*.	0.11	0.33	0.00	0.00	0.00	0.00
*An*. *(Nyssorhynchus) spp*.	0.00	0.00	2.50	0.71	8.50	2.12
*Anopheles spp*.	7.50	1.50	0.00	0.00	2.00	0.00
*Coquillettidia venezuelensis*	267.30	360.40	3.50	2.50	481.00	8.49
*Coquillettidia spp*.	4.00	0.00	0.00	0.00	0.00	0.00
*Culex (Cx) coronator*	0.00	0.00	1.00	0.00	0.00	0.00
*Cx*. *(Cx) declarator*	18.00	3.50	1.00	0.00	3.00	0.00
*Cx*. *(Cx) interrogator*	15.50	14.50	0.00	0.00	0.00	0.00
*Cx*. *(Cx) nigripalpus*	15.00	14.30	0.00	0.00	2.00	0.00
*Culex (Cx*.*) spp*.	198.70	102.40	9.50	3.50	28.00	0.00
*Culex spp*.	0.67	0.00	67.00	20.00	3.00	0.00
*Cx*. *(Anoedioporpa) spp*.	0.67	1.32	0.00	0.00	0.00	0.00
*Culex (Aedinus) spp*.	6.50	3.50	0.00	0.00	0.00	0.00
*Cx*. *(Lutzia) alllostigma*	0.00	0.00	0.00	0.00	3.00	2.83
*Cx*. *(Mel) adamesi*	5.00	0.00	0.00	0.00	0.00	0.00
*Cx*. *(Mel) crybda*	2.00	0.00	0.00	0.00	0.00	0.00
*Cx*. *(Mel) dunni*	72.30	53.90	0.00	0.00	0.00	0.00
*Cx*. *(Mel) ocossa*	2.00	0.00	0.00	0.00	0.00	0.00
*Cx*. *(Mel) pedroi*	24.00	23.50	0.00	0.00	0.00	0.00
*Cx*. *(Mel) spissipes*	23.50	2.50	0.00	0.00	0.00	0.00
*Cx*. *(Mel*) *taeniopus*	15.40	11.50	0.00	0.00	0.00	0.00
*Cx*. *(Mel) vomerifer*	5.00	0.00	0.00	0.00	0.00	0.00
*Cx*. *(Mel) spp*.	177.70	200.30	6.00	5.00	36.00	4.24
*Cx*. *(Mel) spp*. *Secc Mel*	40.00	0.00	0.00	0.00	0.00	0.00
*Deinocerites dyari*	12.00	0.00	0.00	0.00	0.00	0.00
*Haemagogus lucifer*	1.00	0.00	0.00	0.00	0.00	0.00
*Mansonia dyari*	0.22	0.00	1.00	0.00	6.50	0.71
*M*. *indubitans*	19.50	16.50	24.50	0.00	2.00	0.00
*M*. *pseudotitillans*	0.00	0.00	3.00	0.00	0.00	0.00
*M*. *titillans*	0.78	1.64	2.50	0.71	11.00	1.41
*Mansonia spp*.	5.00	0.00	0.00	0.00	0.00	0.00
*Psorophora albipes*	6.00	0.00	0.00	0.00	0.00	0.00
*Ps*. *cingulata*	42.00	33.90	0.00	0.00	0.00	0.00
*Ps*. *confinnis*	29.70	14.00	0.00	0.00	6.50	0.71
*Ps*. *ferox*	3.00	0.00	0.00	0.00	0.00	0.00
*Uranotaenia apicalis*	0.67	2.00	0.00	0.00	0.00	0.00
*U*. *calosomata*	3.50	1.50	4.00	0.00	0.00	0.00
*U*. *geométrica*	0.00	0.00	7.00	1.41	0.00	0.00
*U*. *lowii*	5.30	2.60	0.00	0.00	75.50	7.78
*U*. *pulcherrima*	0.00	0.00	4.00	0.00	0.00	0.00
*Uranotaenia spp*.	84.00	75.00	2.00	0.00	16.00	0.00
*Wyeomyia chalcocephala*	1.00	0.33	0.00	0.00	0.00	0.00
*W*. *hosautos*	1.00	0.00	0.00	0.00	0.00	0.00
*Wyeomyia spp*.	0.00	0.00	1.00	0.00	0.00	0.00
Total Median	1 570.25		142.00		771.00	
Species Richness	52		21		24	

The number of mosquito species and their abundance was variable according to the sampling locality (Table 2 and Table 3). The highest abundance and richness of species was found at Mercadeo where we collected 2787 mosquitoes from 45 species (86.5% of all collected species). This site was followed by Santa Librada (762; 15.1%), Pumona (718; 14.2%), and the lowest mosquito abundance was at Los Pavitos (294; 5.8%), Ciri Grande (281; 5.5%) and El Cacao (215; 4.3%). Alpha diversity, when measured using species richness, decreased in the following order: Mercadeo, Santa Librada, Los Pavitos, Pumona, El Cacao and Ciri Grande. Nevertheless, when the ranking was based on the Margalef index, El Cacao had a larger alpha diversity than Pumona, the rest of locations keeping the same rank (Tables 2 and 3).

**Table 2:** Mosquito species diversity indices for sampling locations in Darién, República de Panamá.

Locations/Index	Mercadeo	Santa Librada	Los Pavitos
Taxa S	45	29	23
Individuals	2919	749	218
Simpson 1-D	0.879	0.772	0.797
Shannon-Wiener H	2.636	2.188	2.185
Margalef	5.264	4.079	3.714

**Table 3:** Mosquito species diversity indices for sampling locations in Panamá and Ngäbe Buglé, República de Panamá.

Province	Panamá		Ngäbe Buglé
Locations/Index	El Cacao	Ciri Grande	Pumona
Taxa S	18	14	20
Individuals	107	146	729
Simpson 1-D	0.773	0.622	0.565
Shannon-Wiener H	2.08	1.551	1.485
Margalef	3.638	2.408	3.186

Regarding mosquito species diversity equity (Tables 2 and 3) we have that according to the Simpson index Mercadeo (0.879), Los Pavitos (0.797) and El Cacao (0.773) were the most diverse. When considering the Shannon-Wiener index sites were ranked as follows: Mercadeo (2.640), Santa Librada (2.188) and Los Pavitos (2,185). The Sorensen similarity index (Table 4) showed that Mercadeo and Santa Librada, in Darién, shared 70% of the mosquito species, the highest species similarity observed in this study. The extent of species similarity was also high between Santa Librada and Los Pavitos (65% of species shared), both located in Darién. Although with a lower species richness, El Cacao and Ciri Grande, both in Panamá, had high mosquito species similarity (58% of species shared), similar to what was observed for Los Pavitos and Pumona (58% of species shared). Interestingly, Pumona shared over half of the species with all other sampling locations but Ciri Grande (40%) (Table 4).

**Table 4:** Mosquito species pairwise Sorensen similarity index for sampling locations from three regions with VEE transmission in the República de Panamá.

Province		Darién		Panamá		Ngäbe Buglé
Sampling Sites	Mercadeo	Santa Librada	Los Pavitos	El Cacao	Ciri Grande	Pumona
Mercadeo	100					
Santa Librada	70	100				
Los Pavitos	56	65	100			
El Cacao	29	30	31	100		
Ciri Grande	25	39	35	58	100	
Pumona	52	56	56	50	40	100

## Discussion

Knowledge about mosquito species diversity in transmission areas is fundamental to understand the entomological risk of vector-borne disease transmission, given that slight bionomic differences between species can lead to significant differences in transmission, the persistence of a disease, or the ability of a vector-borne disease to spread into new host species [36–40]. The mosquito diversity patterns we observed are within what is normally expected for ecological communities of mosquitoes and other diptera species, where local environmental factors are similar [40–43]. Mosquito communities from places geographically close (Fig 1) had more similar faunas, as inferred from the higher Soresen similarity (Table 4). The most species rich region was Darién, followed by Ngäbe Buglé and then by Panamá. Here, it is important to highlight this result likely not only reflects the larger sampling effort at Darién, but also that individual Darién sampling locations had a higher species richness when compared to locations in the other two studied regions. Mercadeo was the sampling site with the highest mosquito abundance and species richness, including most of the *Culex (Melanoconion)* spp, which include many major equine encephalitides vector species [9,44], in contrast with sites from Panamá and Ngäbe Buglé where species from this subgenus were either absent or not identifiable at the species level. This result is very important since it implies a potentially higher entomological risk for enzootic VEEV transmission in Darién, something that could explain the common occurrence of VEE and EEE outbreaks in this region over recent years [4,45]. By contrast Ciri Grande had the lowest species richness, a high abundance of *Culex* spp. (64.2%).

A detailed examination of the species we collected reveals that from the 45 species (out of a total of 52 taxonomic units) we collected, at least 22 species have been reported as VEEV vectors in Panamá or elsewhere in the New World [3,9,26]. The species previously identified as VEEV vectors include 10 *Culex* spp., eight belonging to the *Melanoconium* subgenus, *Spissipes* section: *Cx*. *(Mel) dunni* (Dyar, 1918), *Cx*. *(Mel) pedroi* (Sirivanakam & Belkin, 1980), *Cx*. *(Mel) spissipes* (Theobald, 1903), *Cx*. *(Mel) adamesi* (Sirivanakam & Galindo, 1980), *Cx*. *(Mel) crybda* (Dyar, 1924), *Cx*. *(Mel) vomerifer* (Komp, 1932), *Cx*. *(Mel) ocossa* (Dyar & Knab, 1919) and *Cx*. *(Mel) taeniopus* (Dyar & Knab, 1907); two belonging to the subgenus *Culex*: *Cx*. *nigripalpus* (Theobald, 1901), *Cx*. *declarator* (Dyar & Knab, 1906). Four species belong to the genus *Aedes* subgenus *Ochlerotatus*: *Ae*. *scapularis* (Rondani, 1848), *Ae*. *angustivittatus* (Dyar & Knab, 1907), *Ae*. *serratus* (Theobald, 1901) and *Ae*. *fulvus* (Wiedemann, 1828). Other species of importance for VEEV transmission included: *Coquillettidia venezuelensis*, *Psorophora ferox (*Humboldt, 1819), *Ps*. *albipes* (Theobald, 1907), *Ps*. *confinnis* (Theolbold, 1887), *Mansonia indubitans* (Dyar & Shannon, 1925), *M*. *titillans* (Walker, 1848), *M*. *dyari* (Belkin, Heinemann & Page 1970) and *An*. *pseudopunctipennis* (Theobald 1901). Several of these species are known to have catholic bloodfeeding habits in the República de Panamá [46], an essential condition to facilitate the transmission of enzootic arboviruses [47], and, more generally, a common pattern observed in mosquito communities studied elsewhere [7,48].

The widespread importance of *Culex (Melanoconion)* spp for the transmission of VEEV has been well documented all over Latin America. Specifically, *Cx*. (*Mel*.) *vomerifer*, *Cx*. (*Mel*.) *pedroi* and *Cx*. (*Mel*.) *adamesi* have been found infected with subtype ID in the Magdalena Valley, Colombia [3,47,49,50]. *Cx*. (*Mel*.) *pedroi* has also been found infected with VEEV in Puerto Almendras, Perú [51,52]. *Cx*. *(Mel) taeniopus* is a vector of VEEV subtype IE in México and Central America [9,53]. Similarly, EEEV has been isolated from *Cx*. (*Mel*.) *pedroi* and *Cx*. *(Mel) taeniopus* [54]. *Cx*. *(Mel) vomerifer* from Iquitos, Peru is also susceptible to VEEV [9] and Caraparu virus infection [55,56]. In the República de Panamá *Cx*. *(Mel) aikenii s*. *l*., *Cx*. *(Mel) taeniopus* and *Cx*. *(Mel) vomerifer* have been found infected with VEEV subtype ID [10,53,57]. VEEV has been isolated from *Cx*. (*Mel*) *erraticus*, *Cx*. (*Mel*) *occosa* and *M*. *dyari* in Lake Bayano, Panamá [12,58–60]. *C*. *venezuelensis* is associated with permanent water bodies with floating vegetation [61]. It is a vector of Mayaro, Oropuche, VEE and SLE viruses [33,62,63] and West Nile virus [64]. VEEV has also been isolated from *Ps*. *ferox* and *Ps*. *albipes* [65]. VEEV subtypes IC and IAB have been isolated from *M*. *indubitans*, *M*. *titillans*, *M*. *dyari*, *Ps*. *confinnis* and *An*. *pseudopunctipennis* [13,66,67]. *Ps*. *albipes*, *Ae*. *serratus* and *Ae*. *fulvus* are susceptible to the infection with VEE [13,68]. *Ae*. *angustivittatus* has been found infected with Ilheus virus in Panamá and VEEV in Colombia [69–71]. *Ae*. *scapularis* has been incriminated as VEEV vector in epizootic and enzootic outbreaks[70,72,73]. *Cx*. *nigripalpus* was collected at the three sites in Darién. This species is able to colonize urban and rural landscapes and exhibits a catholic bloodfeeding [74,75]. This species is a major SLE virus vector in the USA [76], but also in Central America, Ecuador and Trinidad and Tobago [77]. *Cx*. *coronator*, also collected in this study at Darién, has an ecology similar to that of *Cx*. *nigripalpus* and has been found infected with SLE virus [71] and Mucambo virus in the Brazilian Amazon [78].

Co-occurring with the VEEV vectors we also found the two most important malaria vector species in the República de Panamá [79,80]: *An*. *(Nys) albimanus* (Wiedemann, 1820), *An*. *(An) punctimacula* (Dyar & Knab, 1906). We also were able to identify several secondary malaria vectors, including: *An*. *pseudopunctipennis*, *An*. *malefactor* **(**Dyar & Knab, 1907), *An*. *neomaculipalpus* (Curry, 1930), *An*. *apicimacula* (Theobald, 1901), *An*. *oswaldoi* (Peryassú, 1922) and *An*. *triannulatus* (Neiva & Pinto, 1922) [80–82]. In general, these malaria vectors were less common than VEEV vectors (Table 1).

A major limitation of our study was our inability to identify a large proportion of *Culex* spp. mosquitoes and other specimens that we were only able to identify at the genus level (29.8%; 1453/4868). This was mostly due to poor specimen conditions, but also to some mosquitoes having distinctive features from those of species described in taxomic keys for mosquito species of the New World. In that sense it would be desirable to develop a barcoding library to molecularly identify all mosquitoes present in the República de Panamá, as has been done elsewhere [83]. This can help to both aid the description of new species and with the identification of morphologically damaged specimens. A second limitation was the sampling during night time, which could have limited the possibility of sampling *Haemagogus* spp, of which we only found one mosquito, and *Sabethes* spp, which we did not collect. Both *Haemagogus* and *Sabethes* are genera with species known to be active during daytime, and which include some species that are medically important, given their role in the transmission of yellow fever virus, another major arbovirus [84,85]. Similarly, the study would have greatly benefited by sampling mosquitoes in areas where no alphavirus transmission has been detected, in order to better understand the role of dominant vector species on disease transmission [38,86] or mosquito diversity on infection [41], while also looking at domestic and wildlife reservoirs, as done for other zoonotic vector borne diseases, for example Leishmaniasis [87–90], in the República de Panamá and for alphaviruses in other regions of Latin America [9].

Finally, we would like to highlight this report is the first to describe the mosquito fauna of locations that have frequently reported VEE outbreaks in the República de Panamá. We were able to identify 22 species that are vectorially competent for VEEV transmission, and other species that also transmit medically important arboviruses and parasites across the New World [9,23,44]. This result is very important as it is a first step for further research looking at the ecology of VEEV-mosquito interactions in order to better understand the enzootic transmission of this and related viruses, especially the invasion of new areas by VEEV [51,91], as well as, transmission during the inter-epizootic periods in the República de Panamá. Further research is needed to better understand why, even though all our study sites had a similar environment, where primary and secondary forest were mixed with cattle farming and agricultural land, in places like the sites in Panamá province there were very few *Culex (Melanoconion)* spp, even though they have been found previously in this region [10,92], and they were common in the two other study regions.
